# Editorial: Oxidative metabolism in inflammation

**DOI:** 10.3389/fimmu.2024.1507700

**Published:** 2024-10-21

**Authors:** Pedro Gonzalez-Menendez, Rosa M. Sainz, Pablo Evelson

**Affiliations:** ^1^ Departamento de Morfología y Biología Celular, School of Medicine, Oviedo, Spain; ^2^ Instituto Universitario de Oncología del Principado de Asturias (IUOPA), University of Oviedo, Oviedo, Spain; ^3^ Instituto de Investigación Sanitaria del Principado de Asturias (ISPA), Hospital Universitario Central de Asturias (HUCA), Oviedo, Spain; ^4^ Facultad de Farmacia y Bioquímica, Departamento de Ciencias Químicas, Cátedra de Química General e Inorgánica, Universidad de Buenos Aires, Buenos Aires, Argentina; ^5^ CONICET, Instituto de Bioquímica y Medicina Molecular (IBIMOL), Universidad de Buenos Aires, Buenos Aires, Argentina

**Keywords:** mitochondria, metabolism, oxidative stress, redox, inflammation, macrophages

Mitochondria are integral to a multitude of cellular functions, including cell proliferation, metabolism, ATP production, and programmed cell death. They play an especially critical role in mediating inflammatory signaling pathways (1). When mitochondria become permeabilized, they can promote inflammation by releasing mitochondrial-derived damage-associated molecular patterns (DAMPs), which are potent triggers of the immune response (2). Furthermore, mitochondria are essential regulators of macrophage activation, differentiation, and survival (3). Alterations in mitochondrial oxidative metabolism significantly affect macrophage polarization: pro-inflammatory macrophages (M1) primarily rely on glycolysis for energy production, whereas anti-inflammatory macrophages (M2) depend on oxidative phosphorylation (OXPHOS) (4).

**Figure 1:**
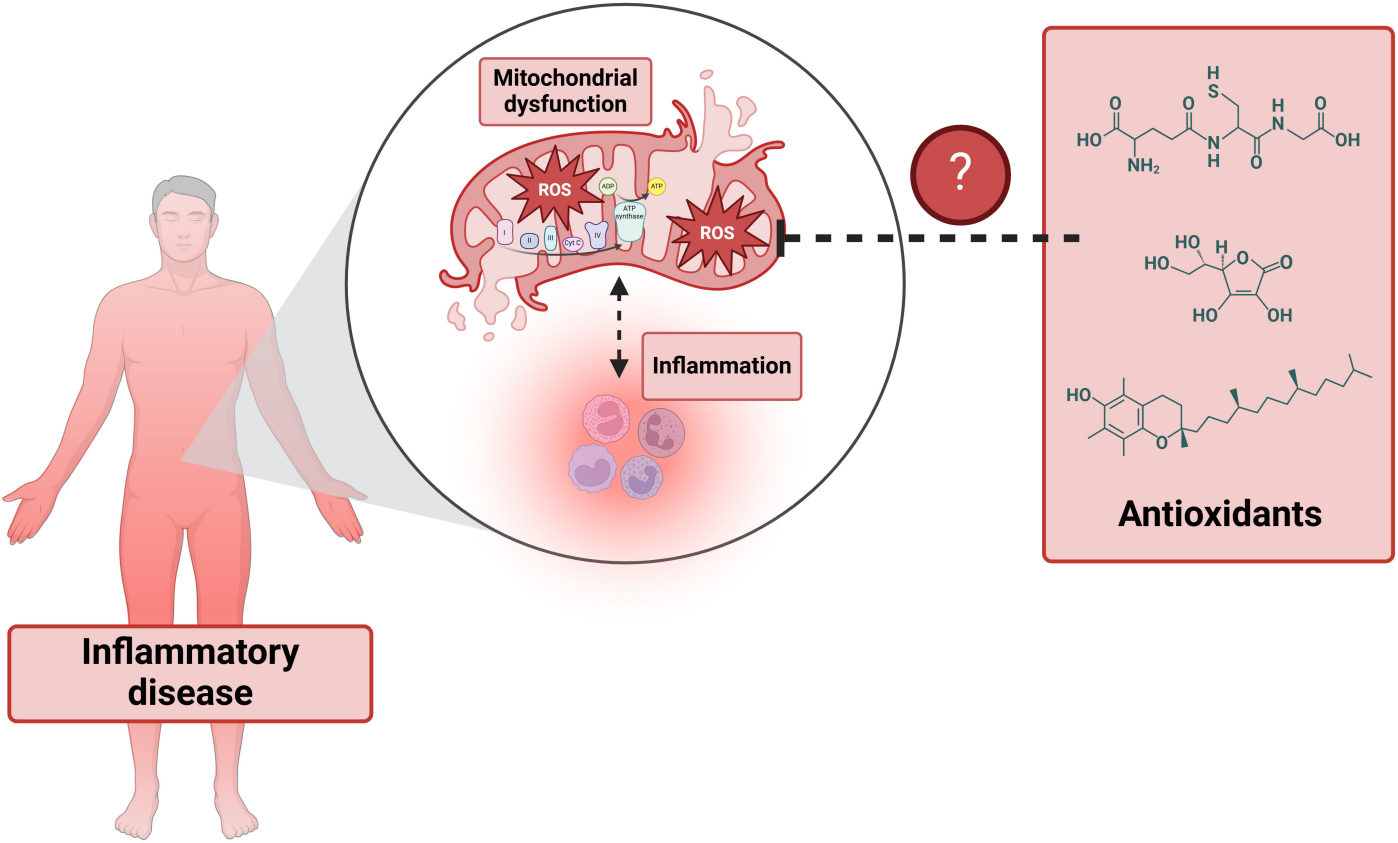
The interplay between inflammation and mitochondrial dysfunction in inflammatory diseases.

In addition to their role in energy metabolism, mitochondria are a major source of reactive oxygen species (ROS), contributing to oxidative stress through electron leakage in the electron transport chain (ETC) and the activity of certain mitochondrial enzymes (5). Oxidative stress is further heightened when macrophages are activated, although these cells possess self-defense mechanisms, such as metabolic reprogramming, to enhance their survival under these conditions (6). An increase in mitochondrial ROS production, or a decrease in antioxidant defenses, can result in significant cellular damage and inflammation. This link between elevated mitochondrial oxidative stress and chronic inflammation is associated with the pathogenesis of various diseases (7), highlighting the importance of mitochondrial function in maintaining cellular homeostasis and immune regulation (**Figure 1**).

Although the intricate relationship between mitochondrial metabolism and the innate immune response is well-established, the therapeutic use of antioxidants has not yielded the expected success/results (8). Moreover, the potential of metabolic reprogramming —whether through altering the metabolic environment or directly targeting mitochondrial function in macrophages for specific inflammatory diseases— remains largely underexplored. The Research Topic “*Oxidative metabolism in inflammation*” therefore aims to investigate whether shifts in oxidative metabolism, mitochondrial reactive oxygen species (ROS) release, mitochondrial membrane potential, and changes in mitochondrial metabolites production/levels influence innate immunity and could serve as viable clinical targets.

This Research Topic comprises 12 articles that span a wide range of themes, from diseases directly linked to inflammation to responses and treatments associated with pathogenic infections, among other related topics. The Research Topic includes six original articles, two brief research reports, two bibliographic reviews, and two mini-reviews.

Sepsis is caused by the body’s extreme response to an infection, leading to widespread inflammation. Thoppil et al. compile a bibliography on the role of the hormone and neurotransmitter norepinephrine in enhancing the anti-inflammatory response by influencing the oxidative metabolism of immune cells. On the other hand, Natalia Rodriguez-Rodriguez et al. summarize how HIV-1 infection inhibits OXPHOS while promoting glycolysis and fatty acid synthesis in immune cells. In original research, Golenkina et al. describe how suppressing leukotriene synthesis in neutrophils with mitochondria-targeted antioxidants, but not thiol-based ones, proves effective against *Salmonella typhimurium* infection. Additionally, Krasic et al. investigate the effects of the anti-inflammatory glucocorticoid methylprednisolone on multisystem inflammatory syndrome associated with COVID-19, noting that it boosts the antioxidant response in erythrocytes. However, patients with low catalase activity who do not respond well to treatment should avoid methylprednisolone, as it may be contraindicated.

Regarding specific inflammatory diseases, osteoarthritis is a degenerative condition characterized by cartilage breakdown, accompanied by increased oxidative stress and an altered inflammatory response (9). Xiong et al. review the effects of the neuroindole melatonin in osteoarthritis, highlighting its ability to reduce inflammation, oxidative stress, and chondrocyte death. Meanwhile, He et al. focus on the use of the diterpenoid alkaloid songorine, which they found to reduce inflammation in osteoarthritis by shifting macrophage polarization from the M1 to M2 phenotype, associated with a metabolic reprogramming towards OXPHOS. Additionally, Zhou et al. propose antioxidant therapy as a novel treatment for chronic rhinosinusitis with nasal polyps, finding that the carotenoid crocin inhibits both M1 and M2 macrophage polarization, reduces the expression of oxidative enzymes NOS2 and NOX1, and enhances the antioxidant capacity of anti-inflammatory M2 macrophages. Similarly, antioxidant therapy may be promising for acute inflammation. Liu et al. demonstrate that the drug troxerutin reduces oxidative stress and inflammation in jellyfish dermatitis by activating the antioxidant Nrf2/HO-1 pathway.

Several chronic diseases are associated with a pro-inflammatory phenotype, including diabetes, which is one of the most common chronic diseases worldwide and is linked to numerous complications due to inflammation (10). Nirenjen et al. have reviewed the role of pro-inflammatory cytokines involved in wound healing, a process that is generally impaired in individuals with diabetes. Furthermore, using single-cell RNA sequencing (RNA-seq), Qi et al. demonstrated that oxidative stress is elevated in peripheral blood mononuclear cells (PBMCs) from patients with ovulation disorders such as polycystic ovary syndrome, primary ovarian insufficiency, and menopause. In these patients, a decrease in naïve CD8 T cells and effector memory CD4 T cells was observed, while there was an increase in natural killer (NK) cells and regulatory NK cells. Non-alcoholic fatty liver disease (NAFLD) is also associated with oxidative stress, as demonstrated by Wang et al. using machine learning and weighted gene co-expression network analysis. They identified that *CDKN1B* and *TFAM* are closely related to oxidative stress in NAFLD.

The Research Topic has also been expanded to include technique articles describing new methods for studying oxidative metabolism in innate immunity. In this regard, Flocke et al. have proposed a non-invasive method to monitor metabolism during inflammation. By using lipopolysaccharide (LPS)-doped Matrigen plugs in mice to induce inflammation, the authors performed ^1^H/^19^F magnetic resonance imaging (MRI) to track the recruitment of ^19^F-labeled immune cells and ^2^H magnetic resonance spectroscopy (MRS) to monitor the metabolic response.

In summary, this Research Topic deepens our understanding of mitochondria-dependent regulation in macrophage biology and explores the role of oxidative metabolism in inflammatory responses associated with chronic diseases and pathogenic organisms. These insights could lead to new clinical strategies for effectively treating acute and chronic inflammation.
